# Assessing Comparative Dimensions of Access When Accessing Primary Care Across Different Levels of Rurality

**DOI:** 10.1111/ajr.70080

**Published:** 2025-08-06

**Authors:** Maddie Higgins, Tiana Gurney, Matthew McGrail

**Affiliations:** ^1^ Rural Clinical School The University of Queensland Brisbane Queensland Australia

**Keywords:** dimensions of access, health service research, healthcare accessibility, modified Monash model, primary care, rural health

## Abstract

**Objective:**

To assess the comparative importance of dimensions of access when accessing primary care across different levels of rurality in Australia.

**Design:**

A quantitative survey using the paired comparison method.

**Setting:**

Regional, rural, and remote communities in Queensland, Australia, are defined by the Modified Monash Model classification.

**Participants:**

3204 households were surveyed, with 192 responses received (6% response rate). After data cleaning, 163 usable surveys were included in the final analysis.

**Main Outcome Measure(s):**

Level of importance for seven dimensions of access: availability, geography, affordability, accommodation, timeliness, acceptability, and awareness.

**Results:**

Awareness was the most important dimension, consistent across all ruralities. Timeliness and availability also ranked highly, though their relative importance varied slightly with the level of rurality. Residents of the regional centre and small rural town ranked timeliness second, while remote and very remote community residents ranked availability second. Geography increased in importance as rurality increased, rising from least important for regional centre residents to mid‐level importance for remote and very remote residents. Affordability consistently ranked low in importance across all ruralities.

**Conclusion:**

This study reveals differences in the importance of dimensions of access when accessing primary care for residents of regional, rural, and remote Australian communities. These findings suggest that strategies to improve primary care access should be tailored to address the most critical factors across different levels of rurality, focusing on improving awareness, availability, and timeliness of primary care services. The increased importance of geography in the remote and very remote community highlights the need for innovative solutions to overcome geographical barriers for these residents.

**Summary**

- What is already known on this subject?
  ○ Access to primary care is crucial for population health, but significant disparities persist between metropolitan residents and rural communities.
  ○ Previous studies have identified various barriers to healthcare access in rural areas, including increased geographical distance, shortage of healthcare providers, and affordability issues.
  ○ While the different dimensions of access have been studied individually, there is a lack of comprehensive comparisons across different levels of rurality using a consistent methodology.
- What does this study add?
  ○ This study contributes to the understanding of how the importance of different dimensions of access varies across regional, rural, and remote communities in Queensland, Australia when accessing primary care.
  ○ It reveals that awareness is consistently the most important dimension of access when accessing primary care across all ruralities, while the importance of geography increases with rurality.
  ○ The findings challenge common assumptions about affordability being a major barrier in rural areas and highlight the need for tailored, community‐specific strategies to improve primary care access in rural contexts.

## 1 Introduction

Access to healthcare plays a vital role in the performance of healthcare systems worldwide [1]. It is one of five key determinants of health, alongside the social and economic environment, the physical environment, and individual characteristics and behaviours [2, 3]. Access to healthcare can be conceptualised as the alignment between healthcare services and community needs [1]. Therefore, adequate access is achieved when healthcare services are well‐suited to the community. This concept aligns with the theory of “the potential ease with which consumers can obtain healthcare at times of need” [4]. Conversely, individuals and communities experience inadequate access to healthcare services when they cannot obtain healthcare during a time of need [5].

Access to primary care is a fundamental contributor to population health [6]. This is commonly delivered by general practitioners (GPs) but incorporates many other clinicians such as nurses, pharmacists, dentists, and allied health professionals delivering community‐based, individual‐level care. Adequate access to primary care is crucial for good health outcomes, with primary care providing accessible, continuous, comprehensive, patient‐focused, and coordinated healthcare [7]. However, significant disparities persist when accessing primary care in regional, rural, and remote Australian communities compared with metropolitan residents. Rural and remote Australian communities face significant healthcare access disparities, with only 67 full‐time equivalent GPs per 100 000 population compared to 114 per 100 000 in major cities [8]. As rurality increases, residents often face higher out‐of‐pocket primary care costs, with average patient contributions reaching $49 per GP visit in remote communities compared to $43 in metropolitan centres [9].

While the concept of access to healthcare, including primary care, has been extensively studied, much of the existing evidence is embedded in metropolitan contexts, where healthcare services are relatively more abundant and nearby [1, 2]. This urban‐centric perspective fails to capture the unique challenges residents face in regional, rural, and remote communities, where access to primary care is often limited and influenced by a complex interplay of individual and geographical factors [10, 11]. A 2013 study by Russell and colleagues comprehensively analysed the critical dimensions of access to healthcare specific to the rural context [4]. This complemented previous key frameworks of Penchansky and Thomas (1981) and Levesque et al. (2013), which were more centred on urban contexts. Consequently, Russell's framework better assists policymakers in addressing barriers to accessing primary care in regional, rural, and remote communities.

The Russell framework comprehensively identifies seven critical dimensions of access that are particularly relevant to regional, rural, and remote communities: availability, geography, affordability, accommodation, timeliness, acceptability, and awareness [4]. Table 1 describes these dimensions further, building on earlier literature across the medical and geography disciplines [2, 12, 13]. However, the relative importance of each dimension across different levels of rurality remains poorly described, hindering the development of targeted policies and interventions to improve primary care access in regional, rural, and remote Australian communities.

**TABLE 1:** Critical dimensions of access to healthcare (adapted from Russell et al. 2013).

Dimension of access to healthcare	Definition
Availability	The volume and types of healthcare services concerning the needs of consumers
Geography	The proximity of healthcare services or healthcare providers to consumers, including how consumers can transcend the distance between their location and healthcare services or healthcare providers
Affordability	The ability of consumers to pay the overall costs of healthcare services, including both direct and indirect costs
Accommodation	The ways resources are organised concerning the ability of consumers to contact, gain entry, and navigate the healthcare system
Timeliness	The extent to which healthcare can be sought, offered or received within a time frame that is optimal for achieving good health outcomes
Acceptability	The attitudes and beliefs of consumers about the healthcare system to the personal and practice characteristics of healthcare providers
Awareness	The sharing of information between healthcare services and consumers and enhancing a consumer's knowledge about the healthcare system

This study aims to assess how these seven dimensions of access vary in importance across different levels of rurality when accessing primary care. It seeks to provide further understanding of the challenges faced by residents in regional, rural, and remote Australian communities.

## 2 Method

A quantitative survey was developed to collect primary data from discrete regional, rural and remote communities (defined by the Modified Monash Model classification). A low‐risk ethics application was approved by the Human Research Ethics Committee. Participants were provided with detailed information about the study's purpose and methods before giving their informed consent to complete the survey. The research sought to quantify the relative importance of dimensions of access from most important to least important when accessing primary care in regional, rural and remote Australian communities. Survey questions were adapted from the Russell framework [4]. The paired comparison method was chosen to simplify complex choices into manageable two‐option comparisons, producing more reliable and precise data while reducing participant cognitive load [14]. Consequently, this method ensured that all respondents directly assessed the importance of each dimension to all other dimensions to enable quantification of the relative level of importance for each dimension.

Respondents assessed the relative importance of each dimension of access by comparing all possible pairs among the seven dimensions, resulting in 21 distinct comparison points. Within the survey, the stem of each pair was phrased as follows: “When visiting a General Practitioner (GP), which one of each of the two pairs is most important to you?”. The specific wording used for each corresponding dimension is summarised in Table 2. A pilot survey was conducted to ensure clarity and effectiveness [15]. Eight community members participated, resulting in minor revisions to the final survey.

**TABLE 2:** Description of each dimension of access in the survey.

Dimension	Description (exact wording)
Availability	The range of services offered by a GP practice
Geography	The distance travelled to visit a GP
Affordability	The cost of visiting a GP
Accommodation	The effort to use services offered by a GP practice
Timeliness	The convenience of appointments with a GP
Acceptability	The respect of your personal or cultural beliefs by a GP
Awareness	The communication of your health needs with a GP

### 2.1 Recruitment

Four communities in rural Queensland, Australia, were selected for the study, including a regional centre, small rural town, remote community and very remote community (the latter two combined to increase response counts). Each community had access to at least one local primary care service with an in‐person general practitioner and was not classified as being ‘nearby’ (< 15 min travel) to larger communities. A random distribution of participation invitations to a subset of households in the regional centre and small rural town was carried out due to their larger population size; in contrast, all households were invited in the remote and very remote communities. An integrated community engagement strategy was implemented for recruitment, utilising print media (local bulletins), online media (emails to local councils and community groups), and collaboration with Primary Health Networks (PHNs) [16]. A paper‐based survey was distributed as a “to the householder” letter via Australia Post including a pre‐paid return envelope. The survey contained a QR code for optional completion online via an electronic version of the survey hosted on Qualtrics [17] to maximise response rates and accommodate varying preferences and technological access among participants [18].

### 2.2 Sampling and Response Rate Considerations

There is extensive literature on survey methodology and response rate optimisation [19, 20, 21, 22]. However, given the known challenges of conducting research in rural communities [23], several strategies were employed to address potential low response rates, including the use of postal distribution to reach widely dispersed communities, offering an incentive (entry to win a $100 gift card) [24], and careful consideration of survey length to balance completeness with participant engagement [25]. However, follow‐up for non‐responders was not possible due to the random distribution method and the anonymity of those responding.

## 3 Results

The total response rate was 6% (192/3204), with 163 fully completed surveys enabling inclusion for the paired comparison analyses. To maintain data integrity, surveys with incomplete paired comparison responses were excluded from the final analysis. This consisted of 108 surveys from a regional centre (66% of fully completed surveys), 37 surveys from a small rural town (23% of fully completed surveys), and 18 surveys (11% of fully completed surveys) from a remote and very remote community.

### 3.1 Regional Centre Participants

**FIGURE 1:**
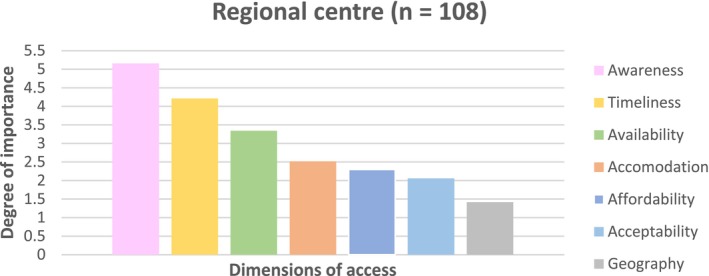
The relative importance of dimensions of access when accessing primary care in a regional centre.

Survey findings from the regional centre revealed the relative importance of the dimensions of access, as shown in Figure 1. Awareness emerged as the most important dimension of access (relative degree of importance above 5.0), followed closely by timeliness (≈4.3) and availability (≈3.4). Accommodation ranked fourth (≈2.5), while affordability and acceptability closely ranked fifth and sixth, respectively (between 2.0 and 2.5). Geography was ranked as the least important dimension of access (≈1.5). These results suggest that for regional centres, prioritising efforts to improve communication of health and health system information, reducing wait times, and increasing service availability would likely have the most significant impact on access to primary care. Affordability and geography appeared less important for the regional community than other dimensions of access.

### 3.2 Small Rural Town Participants

**FIGURE 2:**
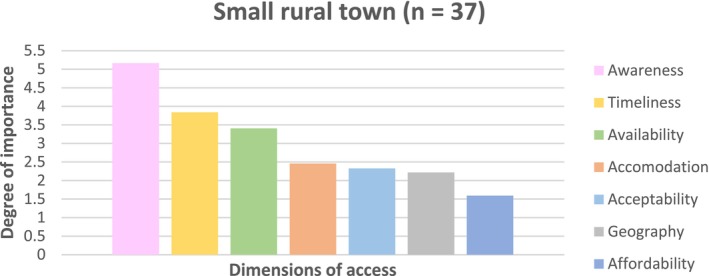
The relative importance of dimensions of access when accessing primary care in a small rural town.

Survey findings from the small rural town revealed the relative importance of the dimensions of access, as shown in Figure 2. Awareness emerged as the most important dimension of access (≈5.0), followed by timeliness (≈3.8) and availability (≈3.4). Accommodation ranked fourth (≈2.5), closely followed by acceptability (≈2.3). Geography was ranked sixth (≈2.2), higher than the regional centre, likely reflecting an increase in rurality. Affordability was ranked as the least important dimension of access (≈1.6). These results suggest that for small rural towns, prioritising efforts to improve communication of health and health system information, reducing wait times, and increasing service availability would likely have the most significant impact on access to primary care. While geography plays a more important role than in the regional centre, affordability appears less important.

### 3.3 Remote and Very Remote Community Participants

**FIGURE 3:**
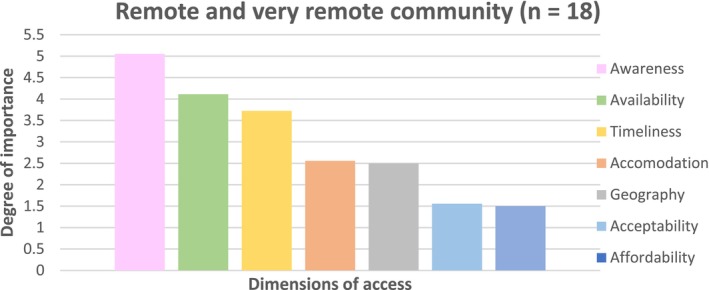
The relative importance of dimensions of access when accessing primary care in a remote and very remote community.

Survey findings from the remote and very remote community revealed the relative importance of the dimensions of access, as shown in Figure 3. Awareness emerged as the most important dimension of access (≈5.0), closely followed by availability (≈4.1) and timeliness (≈3.7). Accommodation and geography were ranked fourth and fifth, respectively (≈2.5), highlighting the increased importance of geographical factors in remote and very remote communities. Acceptability and affordability were ranked the least important dimensions, respectively (≈1.5). These results suggest that for remote and very remote communities, prioritising efforts to improve communication of health and health system information, reducing wait times, and increasing service availability would likely have the most significant impact on access to primary care. Notably, geography plays a more prominent role in remote and very remote communities, while affordability remains less critical. The increased importance of availability in this context likely reflects increased supply challenges in remote and very remote communities.

## 4 Discussion

This study evaluated the comparative importance of dimensions of access when accessing primary care across different levels of rurality in Australia. Findings revealed differences across regional, rural, and remote communities, providing valuable insights for tailoring healthcare policies and interventions to specific rural contexts. However, the low survey response rate necessitates careful interpretation of these results and highlights the broader methodological challenges in rural health research [23].

The findings reveal both commonalities and differences across rurality categories. Awareness consistently emerged as the most important dimension of access when accessing primary care across all ruralities. This suggests that improving (and sustaining) awareness (e.g., how well residents understand their health issues and knowing there are primary care services available within their community for these needs) should be a priority for policymakers and primary care providers, regardless of geographical location [4]. Increasing awareness includes improved health literacy initiatives and ongoing communication with primary care providers to ensure residents' needs are effectively communicated, understood, and supported [1, 4]. The desire for continuing care from GP(s) familiar with their context and health needs is potentially a key reason for this rating [26]. This increase in awareness and the areas that require focus for improvement are relevant when setting and improving health policy. For example, prioritising sustainable, long‐term healthcare workforce models to improve communication of health and health system information and reducing reliance on transient locum services. This approach aims to strengthen residents awareness of both their health conditions and the availability of primary care providers with the appropriate expertise to address their specific and often ongoing needs across different levels of rurality [27, 28].

Timeliness (e.g., primary care services obtained in a timely way) and availability (e.g., sufficient primary care services available within the community) [4] ranked highly across all levels of rurality, though their relative importance varied slightly. Timeliness was ranked second in the regional centre and small rural town, while availability ranked higher than timeliness in the remote and very remote community. These findings suggest that timely and available access to primary care during times of need is crucial for preventing, treating, and managing health conditions, particularly for serious, acute conditions where delayed care can result in poorer health outcomes or increased complications [5, 29, 30]. The shift between rankings in the remote and very remote community reflects the unique challenges of workforce supply that residents in remote and very remote communities face when accessing (readily available) primary care, emphasising the need for tailored healthcare delivery approaches that address specific community needs [10]. From a policy perspective, this may mean ongoing prioritisation of workforce sustainability while considering local transport infrastructure, primary care service distance, and after‐hours availability [27, 28].

Interestingly, affordability (e.g., the ease with which residents can afford primary care services) [4] consistently ranked low in importance across all levels of rurality, contrary to assumptions about affordability being a critical factor when accessing primary care in rural communities [30, 31, 32, 33]. While the quantitative data reveal which dimensions of access are most important relative to each other, they do not explain the underlying reasons for these rankings. Given that a fundamental prerequisite for primary care access is availability, it could be assumed that without existing primary care services, concerns about dimensions such as affordability may become less relevant [1, 2, 34]. Alternatively, the relatively low ranking of affordability may reflect the Australian healthcare context where Medicare provides universal coverage, shifting patient priorities toward dimensions like availability and timeliness rather than affordability [35], despite evidence that rural communities continue to face higher out‐of‐pocket expenses than their metropolitan counterparts [9]. Consequently, this finding warrants further investigation and may suggest that other dimensions of access are perceived as more pressing concerns in regional, rural and remote communities [36, 37].

The importance of geography showed a clear trend, increasing with the level of rurality. This finding aligns with key works that consider geography as a critical barrier when accessing primary care, but likely becomes more prominent with increasingly dispersed populations [13, 38]. Geography was ranked lower in the regional centre, reflecting the proximity of primary care services in regional centres and accessible transport infrastructure [36, 39]. Geography increased to mid‐level importance in the remote and very remote community, reflective of increasing spatial barriers when accessing primary care [38]. From a policy perspective, this may mean ongoing prioritisation of strategic workforce distribution, physical accessibility solutions, alternative digital health models which reduce travel needs, and equitable resource allocation to ensure all communities can access primary care during times of need [27, 28, 40].

The findings of this study have important implications for healthcare policy and resource allocation. Previous research has identified that a one‐size‐fits‐all approach to improving and sustaining access to primary care across different levels of rurality is problematic [37]. Instead, strategies should be tailored to address the most critical dimensions of access in each community, focusing on improving (or maintaining high levels of) awareness, availability, and timeliness of primary care services. For example, sustainable workforce models and appropriate service delivery should be prioritised. This approach should be underpinned by strategic resource allocation that accounts for geographical barriers, ensures equitable access to primary care, and maintains service continuity while considering local community needs and infrastructure limitations [27, 28, 40].

It is important to note the limitations of this study, particularly the low response rates in all communities and small sample size in the remote and very remote community. It is acknowledged that letters addressed ‘to the householder’ do not represent the most practical survey methods, given limited financial resources, this approach was determined to be the most cost‐effective and practical approach for data collection. The low response rate (6%) necessitates caution in generalising these findings. It is also noted that each dimension was represented by a single statement, but collapsing in this way may not be representative of all parts of the dimension. However, this study provides a valuable foundation for understanding the challenges when accessing primary care across different levels of rurality. It offers a starting point for developing targeted interventions and policies to improve primary care access in regional, rural and remote communities.

## 5 Conclusion

This study contributes to our understanding of the comparative importance of dimensions of access when accessing primary care across different levels of rurality in Australia. The findings highlight the need for tailored approaches to improving access to primary care, focusing on awareness across all ruralities and increasing emphasis on geographical accessibility and service availability as rurality increases. The study provides valuable directions for future research and policy development. Rural primary care access hinges on community awareness and strategic workforce planning. Research reveals that rural Queenslanders prioritise locally available and timely primary care services over cost considerations. This highlights the importance of both promoting existing local health services and developing sustainable workforce models tailored to each community's unique geographic and health needs. By focusing on these interconnected factors, healthcare systems can better ensure continuous, accessible primary care across regional, rural, and remote Australian communities. This evidence supports policymakers, healthcare providers, and researchers to address rural Australia's unique challenges and work toward more equitable access to primary care across regional, rural, and remote Australian communities. This study is a crucial step for developing evidence‐based policies and interventions that accurately reflect the needs of these diverse communities.

## Author Contributions

**Maddie Higgins:** conceptualisation; writing – original draft (lead); formal analysis; writing – review and editing. **Matthew McGrail:** formal analysis; writing – review and editing. **Tiana Gurney:** formal analysis; writing – review and editing.

## Ethics Statement

A low‐risk ethics application was approved by the University of Queensland Human Research Ethics Committee (2022/HE000199).

## Data Availability

The data that support the findings of this study are available from the corresponding author upon reasonable request.

## References

[1] J. F. Levesque , M. F. Harris , and G. Russell , “Patient‐Centred Access to Health Care: Conceptualising Access at the Interface of Health Systems and Populations,” International Journal for Equity in Health 12, no. 1 (2013): 18. 23496984 10.1186/1475-9276-12-18 PMC3610159

[2] R. Penchansky and J. W. Thomas , “The Concept of Access: Definition and Relationship to Consumer Satisfaction,” Medical Care 19, no. 2 (1981): 127–140. 7206846 10.1097/00005650-198102000-00001

[3] World Health Organization , Social Determinants of Health (Geneva: World Health Organization, 2024).

[4] D. J. Russell , J. S. Humphreys , B. Ward , et al., “Helping Policy‐Makers Address Rural Health Access Problems,” Australian Journal of Rural Health 21, no. 2 (2013): 61–71. 23586567 10.1111/ajr.12023

[5] Australian Institute of Health and Welfare , Coordination of Health Care: Experiences of Barriers to Accessing Health Services Among Patients Aged 45 and Over (Canberra: Australian Government, 2020).

[6] B. Starfield , L. Shi , and J. Macinko , “Contribution of Primary Care to Health Systems and Health,” Milbank Quarterly 83, no. 3 (2005): 457–502. 16202000 10.1111/j.1468-0009.2005.00409.x PMC2690145

[7] S. Duckett and S. Willcox , The Australian Health Care System 6e EB, 6th ed. (Oxford University Press Australia & New Zealand, 2022).

[8] Department of Health and Aged Care , General Practice Workforce Providing Primary Care Services in Australia (Canberra: Australian Government, 2022).

[9] Australian Institute of Health and Welfare , Medicare Bulk Billing and Out‐Of‐Pocket Costs of GP Attendances Over Time (Canberra: Australian Government, 2022).

[10] L. Bourke , J. S. Humphreys , J. Wakerman , and J. Taylor , “Understanding Rural and Remote Health: A Framework for Analysis in Australia,” Health & Place 18, no. 3 (2012): 496–503. 22418016 10.1016/j.healthplace.2012.02.009

[11] D. Terry , A. Crouch , K. Ervin , K. Glenister , and L. Bourke , “Heterogeneity of Rural Consumer Perceptions of Health Service Access Across Four Regions of Victoria,” Journal of Rural Social Sciences 32, no. 2 (2017): 125.

[12] M. L. Berk and C. L. Schur , “Measuring Access to Care: Improving Information for Policymakers,” Health Affairs (Millwood) 17, no. 1 (1998): 180–186. 10.1377/hlthaff.17.1.180 9455029

[13] M. R. McGrail and J. S. Humphreys , “The Index of Rural Access: An Innovative Integrated Approach for Measuring Primary Care Access,” BMC Health Services Research 9, no. 1 (2009): 124. 19624859 10.1186/1472-6963-9-124 PMC2720961

[14] L. L. Thurstone , “A Law of Comparative Judgment,” Psychological Review 101, no. 2 (1994): 266–270, 10.1037/0033-295X.101.2.266.

[15] E. van Teijlingen and V. Hundley , “The Importance of Pilot Studies,” Social Research Update 35 (2001): 1–4.

[16] Department of Health and Aged Care , Primary Health Networks (Canberra: Australian Government, 2021).

[17] Qualtrics , Qualtrics Provo, Utah, USA: Qualtrics, https://www.qualtrics.com.

[18] D. Leeuw , “To Mix or Not to Mix Data Collection Modes in Surveys,” Journal of Official Statistics 21, no. 2 (2005): 233.

[19] P. Edwards , I. Roberts , M. Clarke , et al., “Increasing Response Rates to Postal Questionnaires: Systematic Review,” BMJ [British Medical Journal] 324, no. 7347 (2002): 1183. 12016181 10.1136/bmj.324.7347.1183 PMC111107

[20] D. A. Dillman , J. D. Smyth , and L. M. Christian , “Internet, Phone, Mail and Mixed‐Mode Surveys: The Tailored Design Method,” Revista Española De Investigaciones Sociológicas 154 (2016): 161–165.

[21] J. R. Evans and A. Mathur , “The Value of Online Surveys: A Look Back and a Look Ahead,” Internet Research 28, no. 4 (2018): 854–887.

[22] J. McPeake , M. Bateson , and A. O'Neill , “Electronic Surveys: How to Maximise Success,” Nurse Researcher 21, no. 3 (2014): 24–26. 24460562 10.7748/nr2014.01.21.3.24.e1205

[23] M. R. McGrail , R. Jones , A. Robinson , C. M. Rickard , M. Burley , and M. Drysdale , “The Planning of Rural Health Research: Rurality and Rural Population Issues,” Rural and Remote Health 5, no. 4 (2005): 426. 16241854

[24] E. Singer and C. Ye , “The Use and Effects of Incentives in Surveys,” Annals of the American Academy of Political and Social Science 645, no. 1 (2013): 112–141.

[25] S. Rolstad , J. Adler , and A. Rydén , “Response Burden and Questionnaire Length: Is Shorter Better? A Review and Meta‐Analysis,” Value in Health 14, no. 8 (2011): 1101–1108. 22152180 10.1016/j.jval.2011.06.003

[26] D. Schwarz , L. R. Hirschhorn , J.‐H. Kim , H. L. Ratcliffe , and A. Bitton , “Continuity in Primary Care: A Critical but Neglected Component for Achieving High‐Quality Universal Health Coverage,” BMJ Global Health 4, no. 3 (2019): e001435. 10.1136/bmjgh-2019-001435 PMC6570977 31263586

[27] Department of Health , National Medical Workforce Strategy 2021–2031 (Canberra: Australian Government, 2021).

[28] Department of Health , Stronger Rural Health Strategy (Canberra: Australian Government, 2021).

[29] F. W. Gardiner , L. Bishop , B. de Graaff , J. A. Campbell , L. Gale , and F. Quinlan , Equitable Patient Access to Primary Healthcare in Australia (Royal Flying Doctor Service of Australia, 2020).

[30] L. Corscadden , J. F. Levesque , V. Lewis , et al., “Barriers to Accessing Primary Health Care: Comparing Australian Experiences Internationally,” Australian Journal of Primary Health 23, no. 3 (2017): 223–228. 27927280 10.1071/PY16093

[31] F. Brundisini , M. Giacomini , D. Dejean , M. Vanstone , S. Winsor , and A. Smith , “Chronic Disease Patients' Experiences With Accessing Health Care in Rural and Remote Areas: A Systematic Review and Qualitative Meta‐Synthesis,” Ontario Health Technology Assessment Series 13, no. 15 (2013): 1–33. PMC3817950 24228078

[32] E. J. Callander , L. Corscadden , and J.‐F. Levesque , “Out‐Of‐Pocket Healthcare Expenditure and Chronic Disease ‐ Do Australians Forgo Care Because of the Cost?,” Australian Journal of Primary Health 23, no. 1 (2017): 15–22. 28442033 10.1071/PY16005

[33] S. Duckett and P. Breadon , Out‐Of‐Pocket Costs: Hitting the Most Vulnerable Hardest (Grattan Institute, 2014).

[34] J. Wakerman , J. S. Humphreys , R. Wells , P. Kuipers , P. Entwistle , and J. Jones , “Primary Health Care Delivery Models in Rural and Remote Australia: A Systematic Review,” BMC Health Services Research 8 (2008): 276. 19114003 10.1186/1472-6963-8-276 PMC2642801

[35] Department of Health , Medicare Benefits Schedule (Canberra: Australian Government, 2022).

[36] B. Ward , J. Humphreys , M. McGrail , J. Wakerman , and M. Chisholm , “Which Dimensions of Access Are Most Important When Rural Residents Decide to Visit a General Practitioner for Non‐Emergency Care?,” Australian Health Review 39, no. 2 (2015): 121–126. 25528572 10.1071/AH14030

[37] S. L. Thomas , J. Wakerman , and J. S. Humphreys , “Ensuring Equity of Access to Primary Health Care in Rural and Remote Australia – What Core Services Should Be Locally Available?,” International Journal for Equity in Health 14, no. 1 (2015): 111. 26510998 10.1186/s12939-015-0228-1 PMC4625941

[38] M. R. McGrail and J. S. Humphreys , “Spatial Access Disparities to Primary Health Care in Rural and Remote Australia,” Geospatial Health 10, no. 2 (2015): 358. 26618314 10.4081/gh.2015.358

[39] T. A. Arcury , J. S. Preisser , W. M. Gesler , and J. M. Powers , “Access to Transportation and Health Care Utilization in a Rural Region,” Journal of Rural Health 21, no. 1 (2005): 31–38. 10.1111/j.1748-0361.2005.tb00059.x 15667007

[40] National Rural Health Alliance , Position Paper: Rural Health Policy in a Changing Climate – Three Key Issues (Canberra: National Rural Health Alliance, 2021).

